# Beyond the hospital gown: a systematic review of the common characteristics and design challenges of patient clothing across healthcare departments

**DOI:** 10.3389/frhs.2026.1858067

**Published:** 2026-07-06

**Authors:** Fida Khader Faraj

**Affiliations:** College of Design and Arts, University of Tabuk, Tabuk, Kingdom of Saudi Arabia

**Keywords:** clothing design, dignity, healthcare design, hospital gown, medical garments, patient clothing, patient experience, patient-Centered care

## Abstract

**Introduction:**

Patient clothing is an essential component of healthcare delivery, influencing clinical practice, patient comfort, dignity, and overall healthcare experience. Despite extensive research on specialized hospital garments, there remains limited understanding of the common design characteristics shared across different hospital departments. This systematic review aimed to identify and synthesize the general and common characteristics of patient clothing across all hospital settings.

**Methods:**

A systematic literature review was conducted following the Preferred Reporting Items for Systematic Reviews and Meta-Analyses (PRISMA) guidelines. Comprehensive searches were performed across major academic databases using predefined search terms. Studies were screened according to established inclusion and exclusion criteria, and methodological quality was assessed before inclusion. Forty eligible studies were included in the final qualitative synthesis.

**Results:**

The review identified four dominant characteristics consistently shaping patient clothing across healthcare settings: clinical accessibility, standardization, infection control, and cost-efficiency. Garments commonly featured loose silhouettes, simplified construction, and open-back or multi-access designs to facilitate clinical examination and treatment. Standardized, one-size-fits-all designs supported efficient distribution, laundering, and reuse across hospital departments. However, these characteristics were consistently associated with poor fit, restricted mobility, inadequate thermal comfort, insufficient body coverage, and limited adaptability to individual patient needs. The evidence further demonstrated that patient clothing frequently overlooks psychological and expressive dimensions, with uniform designs and exposure-related issues contributing to diminished dignity, loss of personal identity, and increased vulnerability.

**Discussion:**

The findings indicate that the defining characteristics of patient clothing are remarkably consistent across hospital departments, reflecting systemic design priorities rather than department-specific requirements. While current designs effectively support clinical efficiency and infection control, they often fail to address patient-centered outcomes. Future patient clothing design should adopt a multidimensional approach that balances functional and clinical requirements with comfort, dignity, personalization, and psychological well-being to enhance the overall healthcare experience.

## 1 Introduction

Patient clothing constitutes a fundamental yet often overlooked component of healthcare environments, positioned at the intersection of clinical functionality, patient experience, and institutional efficiency. Despite its widespread use across all hospital departments—including medical, surgical, emergency, and rehabilitation settings—patient attire has remained largely unchanged over time, raising persistent concerns regarding its adequacy in addressing contemporary healthcare needs. The standard hospital gown, in particular, has been widely criticized for being uncomfortable, exposing, and detrimental to patients' dignity and psychological well-being (1, 2).

The design of patient clothing is inherently complex, as it must accommodate the needs of multiple stakeholders, including patients, healthcare providers, and healthcare systems. From a clinical perspective, garments are primarily designed to facilitate medical procedures by allowing easy and rapid access to the patient's body, often through loose-fitting structures and open-back designs (3, 4). These design features also support infection control protocols, as garments can be easily removed and replaced, minimizing contamination risks and enabling effective hygiene management (5). Consequently, functional accessibility and hygienic performance have historically dominated patient clothing design.

However, recent research highlights that such function-driven approaches often overlook the patient's lived experience. A growing body of literature demonstrates that hospital gowns can negatively influence patients' emotional and psychological states, contributing to feelings of vulnerability, embarrassment, and loss of identity (6). Empirical evidence further indicates that wearing standard hospital attire is associated with reduced perceived control, heightened emotional distress, and lower levels of patient engagement, particularly through its relationship with an external locus of control (7–9). Additional studies show that hospital gowns contribute to feelings of exposure and diminished self-worth, reinforcing passive patient roles within clinical settings (10, 11). These findings suggest that patient clothing may inadvertently function as a barrier to patient-centered care (PCC), limiting patients' sense of autonomy and hindering effective interaction with healthcare providers (1, 6).

From a patient-centered care perspective, clothing plays a crucial role in shaping the patient–provider relationship and influencing patient engagement. PCC emphasizes respect for patients' preferences, autonomy, and active involvement in decision-making processes. However, the standardized hospital gown may unintentionally reinforce power asymmetries between patients and clinicians, symbolizing dependency and reducing patients' sense of agency (10, 11). In this context, patient clothing extends beyond its utilitarian function to become a socio-cultural and psychological artifact that shapes patients' identities and interactions within healthcare settings.

In response, contemporary research increasingly advocates for patient-centered and user-centered design approaches that integrate comfort, dignity, and aesthetic considerations into medical garments (12, 13). These approaches emphasize the importance of involving patients as active stakeholders in the design process and applying frameworks such as the Functional–Expressive–Aesthetic (FEA) model to achieve balanced and holistic solutions (7). At the same time, healthcare systems impose operational and economic constraints—such as cost efficiency, laundering requirements, durability, and logistics—that influence the feasibility and implementation of design innovations (1). As a result, patient clothing reflects a balance between competing priorities: clinical efficiency, patient well-being, and institutional practicality.

Although existing literature has explored various aspects of hospital garments—including functionality, comfort, dignity, and adaptive design—research remains fragmented and often limited to specific clinical contexts. There is a lack of comprehensive synthesis addressing the shared and universal characteristics of patient clothing across different healthcare departments. Identifying these common characteristics is essential not only for advancing theoretical understanding but also for developing unified design frameworks that support more effective, inclusive, and patient-centered clothing systems.

Accordingly, this study aims to systematically examine and synthesize existing research on patient clothing across diverse healthcare settings to identify the general and common characteristics of garments used in different hospital departments, addressing the central research question: *What are the general and common characteristics and preferences of patients' clothing across all hospital departments?* By consolidating findings from multidisciplinary sources, the study provides a comprehensive understanding of recurring design features, challenges, and user needs within patient clothing systems, thereby offering a foundation for future patient-centered design innovations.

To achieve this objective, the study adopts a systematic review methodology guided by the PRISMA framework, ensuring a transparent, rigorous, and replicable process for identifying, analyzing, and synthesizing relevant studies.

## 2 PRISMA

This systematic review follows the PRISMA guidelines, which provide a structured framework to enhance transparency, consistency, and methodological rigor in systematic reviews (14). The application of PRISMA ensures that the processes of study identification, screening, eligibility assessment, and inclusion are conducted and reported systematically, thereby minimizing bias and improving the clarity and reliability of the findings (Liberati et al., 2009). Although originally developed for health-related research, PRISMA has been widely adopted in interdisciplinary fields, including design and textile research, where comprehensive evidence synthesis is required.

In the context of this study, the PRISMA framework was employed to systematically identify and analyze research related to patient clothing across different healthcare departments. This approach enabled the consolidation of fragmented literature spanning medical, design, and psychosocial domains, ensuring a comprehensive understanding of the common characteristics of patient garments. By adhering to PRISMA procedures, the review maintains methodological transparency and provides a replicable process for future research investigating patient-centered clothing design within healthcare systems.

### 2.1 Resources

**Figure 1:**
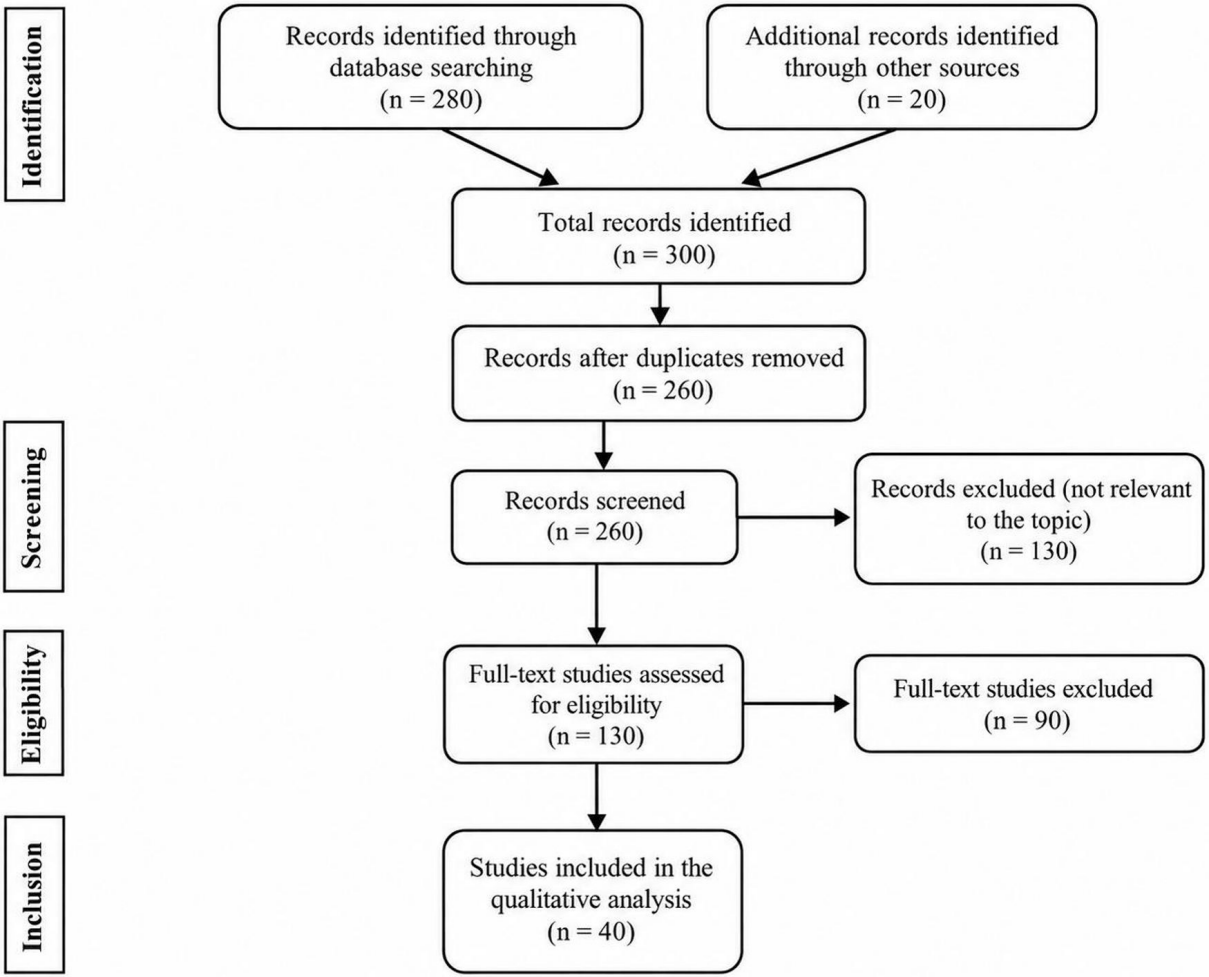
PRISMA (preferred reporting items for systematic reviews and meta-analyses).

A comprehensive electronic literature search was conducted using Scopus, Web of Science (WoS), and Google Scholar to ensure wide coverage of relevant research on patient clothing across healthcare settings (see Figure 1). Scopus served as the primary database for this review, providing access to approximately 24,900 active peer-reviewed journals from 7,000 publishers, covering fields such as healthcare, textile science, design, psychology, and social sciences that are highly relevant to patient clothing, user-centered design, and patient-centered care.

The second database, WoS, comprises approximately 33,000 journals spanning over 256 disciplines, with strong coverage in medical sciences, healthcare research, and interdisciplinary studies. Managed by Clarivate Analytics, WoS also provides a long historical archive with more than a century of citation data, enabling robust identification of influential studies related to hospital garments and patient experience.

In addition to these international databases, Google Scholar was used to capture grey literature and conference proceedings, which are particularly important for emerging themes such as adaptive clothing, medical garment innovation, and user-centered design in healthcare. This approach allowed the review to integrate a broad range of scholarly contributions, providing a comprehensive understanding of the functional, aesthetic, and expressive characteristics of patient clothing across different hospital departments.

### 2.2 Systematic review process

The systematic review process consists of three key stages: identification, screening, and eligibility (see Figure 1).

To minimize selection bias and enhance the reliability of the review process, study screening, eligibility assessment, and quality appraisal were conducted independently by two reviewers.

### 2.3 PICOS framework

The review was guided by the following research question:

What are the general and common characteristics and preferences of patients' clothing across all hospital departments?

To strengthen the transparency of the study selection process, the eligibility criteria were structured according to the PICOS framework (Population, Intervention/Exposure, Comparison, Outcomes, and Study Design), as presented in Table 1.

**Table 1:** PICOS framework applied in the review.

PICOS element	Description
Population (P)	Patients of any age receiving care in healthcare settings, including hospitals, rehabilitation centers, outpatient clinics, and long-term care facilities.
Intervention/Exposure (I)	Patient clothing, hospital gowns, adaptive medical garments, healthcare apparel, and patient-centered clothing designs.
Comparison (C)	Traditional hospital garments, alternative clothing designs, different garment features, or no comparison where comparative analysis was not applicable.
Outcomes (O)	Functional characteristics (comfort, fit, accessibility, mobility, safety, hygiene), aesthetic characteristics (appearance, color, style, visual appeal), expressive characteristics (dignity, identity, autonomy, psychological well-being, patient experience, quality of life).
Study design (S)	Empirical quantitative, qualitative, mixed-methods, design-based, and relevant review studies published in peer-reviewed sources.

### 2.4 Identification

In the initial phase, relevant keywords for the search process were carefully determined in English to ensure comprehensive coverage of international scholarship on patient clothing across healthcare contexts. This stage involved identifying synonyms, related terms, and variations of the primary concepts—such as *patient clothing*, *hospital gown*, *patient attire*, *medical garments*, *clinical apparel*, *adaptive clothing*, *functional clothing*, and *healthcare textiles*—to broaden the scope of the search. Additional keywords were incorporated to capture design-related aspects, including *design characteristics*, *garment design*, *design features*, *clothing performance*, *comfort*, *fit*, *usability*, *accessibility*, *safety*, *aesthetics*, *appearance*, *color*, *pattern*, *identity*, *dignity*, *psychological impact*, *patient experience*, *patient-centered design*, and *user-centered design*. The objective was to equip the selected databases with a comprehensive range of search terms capable of retrieving the maximum number of relevant studies.

The keyword selection process was guided by the research question and refined using academic thesauri, previously used terms in related studies, and automated keyword suggestions provided by Scopus and Web of Science. This ensured conceptual alignment with the multidisciplinary nature of the topic, which spans healthcare, design, and textile research.

The finalized search strategy incorporated Boolean operators, phrase searching, truncation, and field codes to enhance accuracy and coverage. The complete search strings applied across Scopus, Web of Science, and Google Scholar are presented in Table 2.

**Table 2:** Research strings used in database searche*s.*

*Database*	*Search string (Boolean operators, keywords, truncation)*	*Filters applied*
*Scopus*	(“patient clothing” OR “hospital gown*” OR “patient attire” OR “patient wear*” OR “medical garment*” OR “clinical apparel” OR “healthcare garment*” OR “hospital uniform*” OR “adaptive clothing” OR “functional clothing” OR “medical textile*”) AND (“design characteristic*” OR “design feature*” OR “garment design” OR “product design” OR “patient-centered design” OR “human-centered design” OR “design innovation” OR “functional performance” OR comfort OR fit OR sizing OR usability OR accessibility OR mobility OR safety OR hygiene OR “infection control” OR aesthetic* OR appearance OR color OR pattern OR “visual design” OR dignity OR identity OR autonomy OR “psychological impact” OR “emotional response” OR “patient experience” OR “quality of life”)	*Years: 1995–2025; Language: English; Subject Areas: Health Sciences, Social Sciences, Engineering, Design*
*Web of Science (WoS)*	*TS* *=* (“patient clothing preferences” OR “hospital gown*” OR “patient attire” OR “medical garment*” OR “healthcare apparel” OR “adaptive clothing” OR “medical textile*”) AND TS *=* (“design characteristic*” OR “design feature*” OR “garment design” OR “user-centered design” OR “patient-centered design” OR comfort OR fit OR sizing OR usability OR accessibility OR mobility OR safety OR hygiene OR aesthetic* OR dignity OR identity OR “psychological impact” OR “patient experience” OR “quality of life”)	*Timespan: 1995–2025; Language: English*
*Google Scholar*	*allintitle: (“patient clothing” OR “hospital gown” OR “medical garment”) AND (“design” OR “comfort” OR “fit” OR “dignity” OR “identity” OR “patient experience” OR “user-centered design” OR “patient-centered care”) OR (“adaptive clothing” AND healthcare AND design)*	*Years: 1995–2025; First 200–300 results screened*
*Google (General Web Search)*	*(“patient clothing design” OR “hospital gown redesign” OR “medical apparel design” OR “healthcare garment innovation” OR “adaptive medical clothing” OR “patient-centered clothing”) AND (“comfort” OR “dignity” OR “usability” OR “fit” OR “patient experience” OR “quality of life” OR “infection control”)*	*Grey literature, reports, theses, conference papers*

Accordingly, search strings for Scopus, Web of Science, and Google Scholar were developed in January 2026 (see Table 1) after determining all relevant keywords in English. These databases were selected because of their advanced search capabilities, extensive indexing of peer-reviewed journals, rigorous quality control standards, and multidisciplinary coverage, including healthcare, design, textile science, and social sciences. During the identification stage, a total of 280 records were retrieved through database searches conducted in Scopus, Web of Science, and Google Scholar. To enhance the comprehensiveness of the review and minimize publication bias, an additional 20 records were identified through manual searches of reference lists and grey literature sources, including reports, theses, and conference proceedings. Consequently, the identification process yielded a total of 300 records, which were subsequently subjected to screening and eligibility assessment in accordance with the PRISMA framework.

### 2.5 Screening

The second stage of the systematic review process involved screening the records retrieved from the identification stage. After removing 40 duplicate records, 260 unique studies remained for screening. These records were initially assessed based on titles and abstracts, where 130 articles were excluded because they did not meet the inclusion criteria (e.g., not focused on patient clothing, unrelated to healthcare contexts, or lacking relevance to design characteristics and patient experience). Subsequently, 130 full-text articles were evaluated for eligibility (Table 3).

**Table 3:** Inclusion and exclusion criteria.

Criteria	Included studies	Excluded studies
Document type	Empirical research articles, design studies, and relevant review papers	Conference papers, book chapters, book series, and full books
Language	English-language publications	Non-English publications
Study focus	Patient clothing in healthcare settings; hospital garments; medical apparel design; functional, aesthetic, and expressive characteristics; patient experience; user-centered or patient-centered design	Studies unrelated to patient clothing; studies with unclear methodology; studies not addressing healthcare or design-related aspects

### 2.6 Eligibility

At this stage, the full texts of 130 articles were retrieved and carefully reviewed in detail. Each article was examined to verify its alignment with the established inclusion criteria, with particular focus on ensuring that the studies directly addressed patient clothing within healthcare contexts and examined relevant design characteristics, including functional, aesthetic, and expressive dimensions. The evaluation process considered the title, abstract, methodology, and reported findings to determine their relevance to the research objectives. As a result of this process, 90 articles were excluded because they either lacked a clear focus on patient clothing, presented only conceptual discussions without practical or empirical insights, demonstrated insufficient methodological clarity, or reported outcomes not directly related to clothing design or patient experience in healthcare settings.

### 2.7 Quality assessment

To ensure the credibility and methodological rigor of the included studies, a quality appraisal was conducted on the remaining 40 studies, using the Critical Appraisal Skills Programme (CASP) checklist alongside the Joanna Briggs Institute (JBI) appraisal tools. The assessment was carried out to minimize potential bias and ensure consistency in evaluating studies across different disciplines, including healthcare, design, and textile research. Each study was assessed based on several criteria, including research design, clarity of objectives, adequacy of the sample (where applicable), relevance to patient clothing, validity of methods, and the strength and clarity of reported findings. Particular attention was given to whether the remaining studies provided empirical evidence, practical design insights, or clear analytical contributions related to the functional, aesthetic, and expressive characteristics of patient clothing. The quality appraisal indicated that the studies retained for review demonstrated acceptable methodological quality and sufficient relevance to the research objectives. Consequently, the remaining 40 studies met the quality assessment criteria and were included in the final synthesis. These studies were considered sufficiently robust to support the qualitative analysis and to provide a comprehensive understanding of patient clothing characteristics across healthcare departments.

### 2.8 Data extraction and analysis

Following the quality assessment, the remaining studies underwent a systematic data extraction process to capture essential details such as author(s), year of publication, country, healthcare context (e.g., medical, surgical, psychiatric, maternity), study design, sample characteristics (where applicable), and key findings related to patient clothing. Additional emphasis was placed on extracting data concerning design characteristics, including functional performance (e.g., comfort, fit, accessibility, safety), aesthetic aspects (e.g., appearance, color, pattern), and expressive dimensions (e.g., dignity, identity, psychological impact). This process ensured consistency and enabled effective comparison across studies. Once the data were extracted, a thematic synthesis approach was applied, following the integrative review method described by Whittemore and Knafl (45). Through iterative analysis, patterns, similarities, and differences across the studies were identified and systematically organized (Table 4).

**Table 4:** Researched article*s.*

No.	Author(s) and year	Study objective	Key focus
1	Faraj and Mirahan-Zedan (15)	Evaluate user-centered clothing for long-term psychiatric patients	UCD, FEA model, quality of life
2	López-Soto (16)	Identify principles of functional patient-centered gowns	Functionality, dignity, closure systems
3	Punchihewa and Broadbent (17)	Experimentally assess dehumanization caused by gowns	Psychological impact
4	Zhao et al. (18)	Develop emotional response model for gown design	Emotional design (PAD model)
5	Dhanush et al. (19)	Develop antimicrobial surgical gowns	Fabric performance, hygiene
6	Arunachalam and D’Souza (20)	Redesign patient-centered hospital gowns	Privacy, accessibility
7	Syed et al. (1)	Explore stakeholder perspectives on gown design	Functionality, cost, dignity
8	Holder (21)	Investigate dignity among older patients wearing gowns	Privacy, dignity
9	Lorts (13)	Examine patient satisfaction with hospital gowns	Comfort, modesty
10	Frankel et al. (6)	Evaluate a novel gowning system (PALS)	Usability, satisfaction
11	Kam and Yoo (22)	Develop healing-oriented patient clothing	Aesthetic, therapeutic design
12	Hwang et al. (23)	Redesign maternity hospital gowns	Fit, comfort, UCD
13	McAndrews and Brooks (24)	Explore clothing needs of dialysis patients	Functional and emotional needs
14	Morton et al. (8)	Examine impact of gowns on patient well-being	Psychological effects
15	Lucas et al. (25)	Analyze perceptions of gowns among users	Identity, satisfaction
16	Oliver (2)	Critique traditional hospital gown design	Exposure, dignity
17	Jankovska and Park (26)	Evaluate fit and comfort of hospital gowns	Fit, anthropometrics
18	Cogan (9)	Assess gown impact on well-being	Identity, discomfort
19	Diaby et al. (27)	Examine healthcare value frameworks	Patient engagement
20	Bergbom et al. (28)	Explore symbolic role of patient clothing	Identity, anonymity
21	Domecq et al. (29)	Review patient engagement in research	Methodology, participation
22	Wellbery and Chan (11)	Analyze symbolism of medical clothing	Power relations, identity
23	Brasch et al. (30)	Predict preferences for surgical gowns	User preference
24	Black and Torlei (12)	Redesign hospital gowns using UCD	Functionality, dignity
25	Gordon and Guttmann (31)	Develop user-centered gown design	Usability, lifecycle
26	Smith et al. (32)	Evaluate textile impact on pressure ulcers	Fabric performance
27	Topo and Iltanen-Tähkävuori (10)	Study clothing and patient identity	Identity, agency
28	Baillie (33)	Investigate patient dignity in hospitals	Care environment
29	Jha (34)	Analyze caregiver requirements for gowns	Accessibility, usability
30	Cho (35)	Improve satisfaction through gown redesign	Comfort, accessibility
31	Burbidge (36)	Develop improved fastening systems in gowns	Adjustability
32	Simone (37)	Combine gown and robe design	Coverage, comfort
33	Despond et al. (38)	Examine clothing effect on activity and recovery	Mobility
34	Maria (39)	Develop detachable gown design	Modularity
35	Goodwin (40)	Examine role of clothing in patient care	Care practices
36	Jakub (41)	Develop wraparound patient gown design	Structural design
37	Udell (42)	Design adjustable hospital gown	Fastening systems
38	Sperling and Karlsson (43)	Improve clothing fasteners for patients	Ergonomics
39	Sawicki and Herb (44)	Develop functional patient gown design	Accessibility
40	Early patent studies (1988–2000)	Introduce foundational gown innovations	Structural solutions

## 3 Results analysis and discussion

The following section presents an analysis of the main themes derived from the reviewed studies in order to address the central research question: What are the general and common characteristics of patients' clothing across all departments? The thematic analysis of the 40 reviewed studies reveals a set of recurring and interrelated characteristics that define patient clothing across diverse healthcare contexts. These characteristics can be grouped into the following core themes:

### 3.1 Design preferences in patient clothing

Across multiple studies, patients demonstrated a clear preference for designs that balance functionality with familiarity to everyday clothing. Research indicates that traditional hospital garments—particularly the standard backless gown—are widely rejected due to their poor fit, lack of coverage, and institutional appearance, which negatively affect dignity and comfort (8, 16). Patients consistently expressed a desire for clothing that resembles casual or home wear, such as pajama-like garments or two-piece sets, as these enhance psychological comfort and reduce feelings of vulnerability (16, 25).

In terms of fit and sizing, preferences highlight the inadequacy of the “one-size-fits-all” approach. Studies confirm that patients favor garments with adjustable features, improved sizing systems, and designs that accommodate body variability (20, 26). Ill-fitting garments are associated with restricted movement and discomfort, whereas adaptable designs improve mobility and usability.

Patients' preferences regarding structural design elements further reinforce the need for functional yet dignified solutions. Evidence shows a strong inclination toward front or multi-point openings rather than traditional back openings, as these enhance modesty and ease of use (20, 35). In addition, patients favor accessible yet discreet closure systems, such as snaps instead of ties, which maintain dignity while allowing necessary medical access (16). Two-piece garments, including tops and pants, are also widely preferred, as they provide greater flexibility, improved coverage, and a higher degree of independence compared to one-piece gowns (23, 25).

Aesthetic preferences also emerged as a central dimension in patient clothing design. Patients tend to favor visually appealing garments that incorporate colors and patterns inspired by nature, as these elements contribute to relaxation and emotional well-being (18, 22). Furthermore, the inclusion of decorative elements and design variety is preferred over plain, uniform garments, which are often associated with institutional environments and may reinforce negative perceptions of illness (24).

Importantly, design preferences are closely linked to the expressive and psychological functions of clothing. Several studies emphasize that patients prefer garments that preserve personal identity, dignity, and autonomy, rather than reinforcing a passive “sick role” (10, 17). The opportunity to choose between different designs, colors, and styles further enhances patients' sense of control, contributing positively to their emotional well-being and overall experience within healthcare settings (1).

In specialized contexts, such as psychiatric or long-term care, these preferences become even more pronounced. Patients favor clothing that reflects their gender identity and cultural norms, provides adequate modesty without restricting movement, and offers variety and personalization. Such features support emotional renewal, reduce feelings of confinement, and enhance the overall quality of life during extended stays in healthcare facilities (15).

### 3.2 Functional inefficiency of traditional patient clothing

A consistent finding across the literature is that traditional hospital gowns fail to meet fundamental functional requirements, particularly in terms of mobility, fit, and usability. Standardized “one-size-fits-all” designs restrict movement, complicate daily activities, and fail to accommodate body variability, especially among patients experiencing physiological changes (13, 24, 26). These limitations are further exacerbated in long-term care contexts, where prolonged use intensifies discomfort and functional inadequacy (15).

From a clinical perspective, caregivers also report that conventional gowns hinder medical procedures due to poor accessibility and inefficient design features (34). Studies emphasize that functional requirements must simultaneously address patient comfort and clinical accessibility, highlighting the dual-user nature of medical garments (1, 20). The inadequacy of traditional designs is further confirmed by comparative evaluations, which demonstrate that redesigned gowns significantly improve usability and performance (16, 35).

Material selection also plays a crucial role in functional performance. Fabrics lacking breathability and moisture absorption contribute to discomfort and skin irritation, particularly in long-term use (19, 32). Conversely, blended materials and advanced textiles enhance hygiene, durability, and physiological comfort (46, 47).

### 3.3 Psychological impact and dehumanization

A dominant theme across studies is the profound psychological impact of hospital clothing, particularly its role in reinforcing vulnerability, loss of control, and dehumanization. Empirical evidence demonstrates that wearing hospital gowns increases feelings of inferiority, distress, and reduced agency (8, 17). Patients frequently associate gowns with the “sick role,” which symbolically reinforces illness identity and dependence (9, 10).

The symbolic dimension of clothing extends beyond individual perception to influence social interactions and institutional dynamics. Hospital gowns contribute to hierarchical relationships between patients and healthcare providers, reinforcing power imbalances (11). This aligns with findings that gowns function as tools of institutional control, promoting passivity and compliance (28).

Qualitative studies further reveal that exposure, lack of privacy, and uniformity diminish dignity and self-esteem (2, 33). Patients report feelings of embarrassment and identity loss, particularly when garments fail to provide adequate coverage (25). Importantly, experimental evidence confirms that these psychological effects are not merely subjective but measurable. Patients wearing gowns exhibit higher levels of perceived dehumanization compared to those wearing personal clothing (17). This underscores the critical role of clothing as a determinant of psychological well-being in healthcare environments.

### 3.4 Aesthetic deficiency and its psychological consequences

Aesthetic aspects of patient clothing are frequently overlooked, despite their significant impact on psychological well-being. Traditional gowns are characterized by poor visual appeal, lack of variety, and absence of personalization, contributing to negative self-perception (16, 22).

Patients consistently express a desire for clothing that resembles everyday attire, emphasizing the importance of appearance in maintaining self-esteem and normalcy (15, 25). Uniform and gender-neutral designs further exacerbate dissatisfaction, particularly among female patients who seek clothing aligned with their identity and cultural expectations. Research demonstrates that incorporating aesthetic elements such as color, pattern, and silhouette can positively influence mood and emotional stability (18). Natural motifs and visually appealing designs are associated with relaxation and psychological comfort (48, 49).

### 3.5 Fabric characteristics

Fabric characteristics emerged as a central theme influencing both the functional performance and psychological comfort of patient clothing. Across the reviewed studies, fabric properties such as softness, breathability, moisture management, elasticity, durability, and antimicrobial performance were consistently identified as critical determinants of user satisfaction and well-being. In the current study, the inadequacy of existing fabrics—particularly their poor moisture absorption and contribution to skin irritation—was evident, aligning with prior findings that conventional hospital textiles often lack sufficient softness and air permeability, thereby restricting thermal regulation and comfort (1, 26). Similarly, Smith et al. (32) demonstrated that advanced synthetic fabrics designed to reduce friction, moisture, and shear significantly lowered the incidence of pressure ulcers, highlighting the clinical importance of appropriate textile selection.

The literature further emphasizes the advantages of blended fabrics that combine natural and synthetic fibers. Natural fibers such as cotton and bamboo enhance softness, breathability, and moisture absorption, while synthetic components improve durability, elasticity, and resistance to repeated laundering (19) (46). For instance, bamboo–polyester blends have been shown to exhibit antimicrobial properties, contributing to improved hygiene and infection control in medical settings (19). These findings are consistent with the outcomes of the present research, where the proposed cotton–lycra blend significantly improved tactile comfort, reduced skin irritation, and enhanced overall user satisfaction. In addition to physical comfort, fabric characteristics also play a role in psychological well-being. Soft textures and thermally comfortable materials contribute to a sense of security and relaxation, whereas rough or non-breathable fabrics may exacerbate discomfort and emotional distress (8).

### 3.6 Identity, expression, and symbolism in patient clothing

Clothing functions as a medium of self-expression and identity construction, making its role in healthcare settings particularly significant. Studies indicate that hospital clothing often suppresses individuality, leading to identity loss and reduced self-worth (10, 28). Patients describe the transition to hospital clothing as a symbolic shift into the patient role, marked by reduced autonomy and social status (11). This is particularly pronounced in psychiatric settings, where clothing reinforces stigma and institutional identity (15). Expressive elements such as color and design play a crucial role in restoring identity and emotional balance. Research using the PAD (Pleasure–Arousal–Dominance) model demonstrates that clothing attributes directly influence emotional responses and behavioral outcomes (18). Similarly, color preferences are shaped by personal experiences, cultural context, and psychological states (50, 51).

### 3.7 Modesty, privacy, and cultural considerations

Modesty and privacy are central to patient dignity and satisfaction, particularly in culturally sensitive contexts. Studies consistently highlight that inadequate coverage and exposure are major sources of discomfort and distress (6, 35). Traditional gowns often fail to maintain privacy during movement or medical procedures, leading to embarrassment and reduced confidence (2). Redesign efforts that incorporate full coverage, layered garments, and alternative structures significantly improve patient experience (16, 20).

## 4 Discussion

The findings of this thematic analysis provide a comprehensive understanding of patient clothing within healthcare settings, highlighting a persistent misalignment between traditional hospital garments and contemporary patient-centered care principles. Across the reviewed studies, patient clothing emerges not merely as a functional medical requirement but as a multidimensional component influencing physical comfort, psychological well-being, identity, and overall healthcare experience (1, 10, 28).

A central outcome of this review is the consistent rejection of traditional hospital gowns, particularly the standard backless design. Evidence across multiple studies demonstrates that these garments fail to satisfy basic functional, aesthetic, and expressive needs (2, 8, 16). Patients frequently report discomfort, inadequate coverage, and restricted mobility, all of which undermine the intended purpose of facilitating care (13, 26). At the same time, the institutional appearance of such garments reinforces feelings of vulnerability and loss of dignity (25, 33). These findings align with broader healthcare shifts toward patient-centered care, emphasizing that clothing should support, rather than compromise, patient well-being (1, 20).

The analysis further confirms that patients' design preferences are strongly oriented toward familiarity and normalcy. Garments resembling everyday clothing—such as two-piece sets or pajama-like designs—are consistently preferred due to their ability to reduce psychological distress and enhance comfort (16, 23). This preference reflects a deeper need to maintain continuity with one's identity outside the hospital environment (10). In this context, clothing functions as a stabilizing element that mitigates the disruptive nature of hospitalization. The inadequacy of standardized sizing systems further reinforces this issue, as ill-fitting garments not only hinder movement but also exacerbate discomfort and dissatisfaction (24, 26). Adjustable and adaptive designs therefore represent a critical direction for improving both usability and inclusivity (20).

From a functional perspective, the dual-user nature of patient clothing—serving both patients and healthcare providers—introduces inherent design challenges. While clinical accessibility remains essential, the findings indicate that current designs often prioritize provider convenience at the expense of patient dignity and comfort (6, 34). This imbalance highlights the necessity of integrated design approaches that equally consider both user groups (1). Innovations such as multi-point openings, modular structures, and improved closure systems demonstrate the potential to reconcile these competing requirements (20, 35), suggesting that functionality and dignity are not mutually exclusive but can be achieved simultaneously through thoughtful design (12).

Material selection also emerges as a key determinant of both functional performance and patient experience. The reviewed studies consistently emphasize the importance of breathable, soft, and moisture-regulating fabrics in enhancing comfort and preventing adverse clinical outcomes such as skin irritation and pressure ulcers (1, 32). The growing use of blended and advanced textiles reflects an increasing recognition of the role of fabric technology in healthcare environments (19). Importantly, these findings support the argument that textile innovation should be considered a fundamental component of patient clothing design rather than a secondary consideration (26).

Beyond physical functionality, the psychological and symbolic dimensions of patient clothing represent one of the most significant contributions of this review. The literature clearly demonstrates that hospital garments play a critical role in shaping patients' emotional states and social identities (8, 9). The association of hospital gowns with the “sick role” contributes to feelings of dependency, loss of control, and dehumanization (10, 17). This symbolic function extends to the broader institutional context, where clothing reinforces hierarchical relationships between patients and healthcare providers (11). Such findings underscore the importance of re-evaluating hospital clothing not only as a practical tool but also as a cultural artifact embedded within healthcare systems (28).

Aesthetic considerations, often overlooked in medical garment design, are shown to have substantial psychological implications. The absence of color, pattern, and design variation contributes to negative self-perception and emotional distress (22, 25), whereas visually appealing garments can promote relaxation and improve mood (18). The preference for nature-inspired designs and varied aesthetics reflects the broader role of environmental and sensory factors in healing processes (22). These findings support the integration of aesthetic dimensions within the FEA (Functional–Expressive–Aesthetic) model, reinforcing that visual design is integral to patient-centered outcomes (15).

Issues of identity and self-expression further highlight the limitations of current patient clothing systems. The transition from personal clothing to standardized hospital garments represents a symbolic loss of individuality and autonomy (10, 28). This effect is particularly pronounced in long-term and psychiatric care settings, where clothing significantly influences patients' sense of self and social participation (15). Providing options for personalization, including variations in color, style, and design, can therefore play a critical role in restoring agency and supporting psychological well-being (1).

Modesty and privacy emerge as universally significant concerns across all healthcare contexts. Studies consistently highlight that inadequate coverage and exposure are major sources of discomfort and distress (2, 35). The consistent inadequacy of traditional gowns in providing sufficient coverage indicates a systemic design flaw rather than a context-specific issue (16). Redesign strategies that incorporate full coverage, layered structures, and culturally sensitive features demonstrate clear improvements in patient satisfaction (6, 20).

Importantly, the comparative analysis across hospital departments reveals a high degree of consistency in the fundamental characteristics of patient clothing. Regardless of clinical specialization, garments are shaped by shared requirements related to accessibility, hygiene, standardization, and cost-efficiency (1, 30). However, these common characteristics often come at the expense of personalization, comfort, and psychological well-being (25). The persistence of such limitations across departments suggests that the challenges associated with patient clothing are systemic rather than isolated, requiring comprehensive and interdisciplinary solutions.

Although the review identified several common characteristics and preferences across healthcare settings, the findings should be interpreted with consideration of the specific clinical context in which patient clothing is used. Clothing requirements may vary substantially across hospital departments due to differences in clinical procedures, patient mobility, length of stay, and psychosocial needs. For example, garments used in surgical units often prioritize rapid clinical access, infection control, and procedural efficiency, whereas clothing in psychiatric, rehabilitation, and long-term care settings may place greater emphasis on dignity, identity, autonomy, comfort, and social participation. Similarly, intensive care, maternity, pediatric, and oncology departments may require specialized design features tailored to their unique patient populations and care requirements. Therefore, while the present review identified overarching themes and common design characteristics, these findings should not be interpreted as uniformly applicable across all healthcare departments. Future research should investigate department-specific clothing requirements to better understand how functional, aesthetic, and expressive needs vary across clinical contexts.

Finally, the increasing adoption of user-centered design approaches and the FEA model signals a paradigm shift in the development of medical garments. Contemporary research emphasizes the need to balance functional efficiency with expressive and aesthetic considerations, positioning patient clothing as an integral component of holistic healthcare design (12, 23). The convergence of evidence across the 40 high-quality studies included in this review provides strong support for rethinking hospital garments as adaptive, patient-centered systems that enhance both clinical outcomes and human experience.

## 5 Findings

### 5.1 Patients clothing characteristics

Across healthcare departments, patient clothing demonstrates a consistent set of general and common characteristics shaped primarily by clinical priorities, institutional constraints, and, to a lesser extent, patient-centered considerations. First, garments are predominantly designed to ensure clinical accessibility and infection control, often through loose silhouettes, simplified structures, and open or multi-point access features that facilitate examination and treatment (19, 20, 23). Second, standardization and limited sizing systems are widely implemented to support efficiency in distribution, laundering, and reuse; however, this approach frequently results in poor fit, discomfort, and lack of adaptability to diverse body types (13, 26). Third, patient clothing across departments consistently exhibits functional limitations, including restricted mobility, difficulty in independent use, and incompatibility with patients' physical and medical needs, particularly among those with chronic conditions (24, 34). Fourth, a major recurring characteristic is the inadequate provision of dignity and privacy, as open-back designs, insufficient coverage, and inappropriate fastening systems contribute to feelings of exposure, vulnerability, and embarrassment (2, 9, 16).

In addition, patient clothing is characterized by limited aesthetic and expressive value, with uniform, institutional designs that lack variation in color, pattern, and style, thereby diminishing patients' sense of identity and self-expression (25, 28). This uniformity reinforces the symbolic role of clothing in healthcare, where garments often function as markers of illness, dependency, and the “sick role,” contributing to reduced perceived control and psychological distress (8, 17). Furthermore, patient clothing reflects a dual-user design requirement, as it must simultaneously meet the needs of patients (comfort, modesty, psychological well-being) and healthcare providers (efficiency, accessibility, usability), often resulting in a design imbalance that prioritizes clinical convenience over patient experience (12, 34). Despite these limitations, emerging evidence highlights a growing shift toward user-centered and adaptive design approaches, incorporating improved coverage, adjustable features, enhanced materials, and aesthetically informed elements to support both functional performance and emotional well-being (6, 22, 35).

Overall, the common characteristics of patient clothing across all departments can be summarized as a system dominated by functionality, standardization, and efficiency, yet consistently challenged by shortcomings in fit, comfort, dignity, identity, and personalization, indicating the need for a more balanced, patient-centered design paradigm.

Although several common characteristics were identified across healthcare settings, the specific design requirements of patient clothing vary according to the clinical context. For example, garments used in surgical departments often prioritize rapid clinical access, infection control, and procedural efficiency, whereas clothing in psychiatric, rehabilitation, and long-term care settings may place greater emphasis on safety, dignity, autonomy, identity, and social participation. Similarly, specialized requirements exist in maternity, pediatric, oncology, and intensive care settings. Therefore, the characteristics identified in this review should be interpreted as overarching patterns rather than uniformly applicable features across all hospital departments.

### 5.2 Patients’ preferences

The reviewed studies reveal a clear and multidimensional set of preferences for patient clothing, structured around functional, aesthetic, and expressive requirements (26). From a functional perspective, patients consistently prefer garments that facilitate ease of movement, physical comfort, and adaptability to daily activities (15). Current designs are widely criticized for restrictive cuts, poor fit, and inflexible structures that hinder movement during essential activities such as sleeping, walking, and self-care (9). Accordingly, patients express a strong preference for looser silhouettes, flexible construction, and ergonomically designed openings that enhance mobility and independence (20, 35). In addition, adjustable and inclusive sizing systems are essential, as standardized “one-size-fits-all” approaches fail to accommodate body variability and treatment-related changes (24). Patients favor garments that provide proportional fit in both length and width, avoiding the trade-off between tightness and excessive looseness (15).

Fabric-related preferences further emphasize the importance of softness, breathability, and moisture management, as poor-quality textiles are associated with discomfort, skin irritation, and reduced thermal comfort (19, 32). Patients therefore prefer fabrics that are gentle on the skin, hygienic, and suitable for prolonged wear (19). At the same time, safety considerations remain important, particularly in psychiatric contexts, where clothing must balance durability and risk reduction without compromising comfort (15).

From an aesthetic perspective, patients demonstrate a clear preference for clothing that is visually appealing, gender-sensitive, and non-institutional (22). Uniform and plain designs are consistently rejected, as they resemble institutional or prison-like attire and undermine individuality (2026) (2). Instead, patients favor garments that resemble everyday clothing, incorporating decorative elements, varied styles, and culturally appropriate forms (16). Color and pattern preferences are also significant, with patients favoring vibrant, nature-inspired designs that promote comfort and positive emotional responses (18).

The expressive dimension further highlights patients' desire for clothing that supports identity, dignity, and autonomy (28). Current garments are often perceived as symbols of illness and loss of self, reinforcing dependency and the “sick role” (8, 9). Consequently, patients prefer clothing that resembles normal attire and allows them to feel natural rather than institutionalized (25). Maintaining modesty and cultural appropriateness is also a critical requirement, particularly in culturally sensitive contexts (6, 35). Furthermore, patients emphasize the importance of choice and variation, expressing a need for multiple clothing options in terms of style, color, and design, as this enhances autonomy and emotional well-being (1, 15).

Patient preferences also demonstrate important variations across healthcare departments and patient populations. While comfort, dignity, privacy, and appropriate fit emerged as common preferences, the relative importance of specific design features differed according to clinical needs. For instance, psychiatric patients often place greater emphasis on safety-related clothing characteristics, whereas surgical patients may prioritize ease of examination and treatment access. Likewise, long-term care patients may value personalization and identity expression more strongly than patients undergoing short-term procedures. These differences suggest that patient preferences should be considered within their specific healthcare context when developing or evaluating patient clothing systems.

## 6 Conclusion

This study provides a comprehensive synthesis of the literature on patient clothing, demonstrating that hospital garments constitute a critical yet often overlooked component of healthcare design. The findings confirm that traditional patient gowns—particularly the standard backless design—are fundamentally misaligned with contemporary patient-centered care principles. Across the reviewed studies, these garments consistently fail to meet essential functional, aesthetic, and expressive requirements, negatively affecting patient comfort, dignity, and overall healthcare experience.

The analysis highlights that patient clothing should no longer be understood solely as a utilitarian medical necessity but rather as a multidimensional system that directly influences physical performance, psychological well-being, and identity construction. Evidence indicates that patients prefer garments that resemble everyday clothing, offer improved fit and adjustability, and incorporate aesthetically pleasing elements. Such preferences reflect a broader need to preserve autonomy, dignity, and continuity of self within the healthcare environment.

From a functional perspective, the dual-user nature of patient clothing—serving both patients and healthcare providers—necessitates a balanced design approach. While clinical accessibility remains essential, it should not compromise patient privacy or comfort. The findings demonstrate that innovative design solutions, including modular structures, improved closure systems, and adaptive sizing, can successfully reconcile these requirements.

Material selection also emerges as a key determinant of garment performance and user satisfaction. Advanced and blended textiles contribute significantly to comfort, hygiene, and clinical outcomes, underscoring the importance of integrating textile innovation into medical garment design. At the same time, the psychological and symbolic dimensions of patient clothing—particularly its association with vulnerability, dependency, and the “sick role”—highlight the need for a fundamental rethinking of its role within healthcare systems.

The study further reveals that many of the limitations of patient clothing are systemic and consistent across hospital departments, rather than context-specific. Standardization, cost-efficiency, and infection control continue to dominate design priorities, often at the expense of personalization, comfort, and emotional well-being. This indicates the necessity of adopting interdisciplinary and user-centered design frameworks, such as the FEA model, to achieve more holistic and effective solutions.

In conclusion, patient clothing should be repositioned as an integral component of healthcare quality and patient experience. Future design approaches must prioritize flexibility, inclusivity, and cultural sensitivity, while simultaneously addressing functional and clinical requirements. Advancing patient-centered garment design has the potential to enhance not only comfort and efficiency but also dignity, identity, and overall quality of care within healthcare environments.

## Data Availability

The original contributions presented in the study are included in the article/Supplementary Material, further inquiries can be directed to the corresponding author.
